# Vitrectomy for Epiretinal Membrane Peeling in Patients with Diabetic Retinopathy—Functional and Morphological Outcome

**DOI:** 10.3390/jcm14145128

**Published:** 2025-07-18

**Authors:** Patricia Hülse, Sarah Janott, Verena Schöneberger, Claudia Brockmann, Thomas A. Fuchsluger, Friederike Schaub

**Affiliations:** 1 Department of Ophthalmology, University Medical Center Rostock, 18057 Rostock, Germanyfriederike.schaub@med.uni-rostock.de (F.S.); 2 Department of Ophthalmology, Medical Faculty, University Hospital Düsseldorf, Heinrich Heine University Düsseldorf, 40225 Düsseldorf, Germany

**Keywords:** diabetic retinopathy, epiretinal membrane peeling, vitrectomy

## Abstract

**Background**: Secondary epiretinal membrane (ERM) is a common complication of diabetic retinopathy, but data on surgical outcome is limited. The aim of this study was to evaluate anatomical and functional outcomes after pars plana vitrectomy with ERM peeling in eyes with diabetic retinopathy. **Methods**: A retrospective analysis was conducted on 87 eyes of 87 consecutive patients with diabetic retinopathy who underwent ERM peeling over a ten-year period (04/2013–11/2022). Collected data included demographics, best-corrected visual acuity (BCVA), stage of diabetic retinopathy, and optical coherence tomography parameters such as central subfield retinal thickness (CSRT), macular volume (MV), and presence of hyperreflective foci, subretinal fluid, and intraretinal fluid. Statistical analyses were performed using a paired *t*-test and the Wilcoxon test. **Results**: The majority of patients had type 2 diabetes (96.6%), and 69.0% presented with diabetic macular edema (DME). The mean follow-up was 2.2 ± 2.0 years. Significant postoperative reductions were observed in CSRT (from 377.20 ± 99.28 µm to 337.99 ± 113.834 µm; *p* = 0.008) and MV (from 10.11 ± 1.46 mm^3^ to 99.28 ± 1.07 mm^3^; *p* < 0.001). No significant changes in BCVA were observed across the entire study cohort. ERM recurrence was rare (2.3%), and no major complications occurred. **Conclusions**: ERM peeling in diabetic eyes leads to significant anatomical improvement, especially in advanced diabetic retinopathy and DME, but with limited functional gains. The surgical indication should be carefully considered.

## 1. Introduction

Epiretinal membrane (ERM) is a common retinal condition that can significantly impact visual function [1]. While ERMs may occur primarily following complicated posterior vitreous detachment, they can also develop secondarily to various retinal pathologies, including diabetic retinopathy (DR) [2]. The formation of ERMs in diabetic patients is attributed to the accumulation of advanced glycation end products and increased levels of inflammatory mediators [3]. The prevalence of ERMs in diabetic populations varies widely, with reports ranging from 6.5% to 33.3% [4].

These membranes can act as physical barriers, potentially reducing the efficacy of intravitreal treatments for diabetic macular edema (DME) by impeding drug penetration [5]. Moreover, ERMs may contribute to the progression of DME through the production of cytokines and growth factors that stimulate pathogenic neovascularization [5]. Recent studies have investigated the outcome of surgical intervention for ERMs in diabetic patients. Pars plana vitrectomy (PPV) with ERM peeling has demonstrated anatomical benefits, including reduced central macular thickness, and has shown potential for visual improvement in diabetic patients with and without DME [4,6]. Furthermore, diabetes, as a complex systemic condition, can affect both the natural course of retinal disease and surgical outcomes. Combined with the presence of systemic and ocular comorbidities and the inherent risks of surgery, these uncertainties make the decision for surgical intervention in diabetic patients particularly challenging and necessitate individualized evaluation. Therefore, further investigation is warranted for a better understanding of visual and anatomical outcomes after ERM peeling in this patient population. In the present study, we aim to evaluate functional changes as well as morphological parameters such as central retinal thickness and macular volume in diabetic patients who underwent PPV with ERM peeling.

## 2. Materials and Methods

### 2.1. Study Settings

In this monocentric, retrospective study, we reviewed data for consecutive patients who were diagnosed with diabetes and underwent PPV for ERM peeling at the Department of Ophthalmology of the University Medical Center Rostock between April 2013 and November 2022. The study complies with the ethical principles for medical research as outlined in the Declaration of Helsinki, and approval was given by the local ethics committee of the University Medical Center Rostock (IRB No. A 2022-0124).

This study included patients with mild, moderate, or severe non-proliferative diabetic retinopathy (NPDR) and those with proliferative diabetic retinopathy (PDR). The grading of DR stages followed the criteria outlined in the International Classification of Diabetic Retinopathy staging scale as previously defined [7]. In the 30 cases where both eyes per patient fulfilled the eligibility criteria, only the eye operated on first was selected to prevent inter-eye correlation bias. We searched our database for all patients with diabetic retinopathy who underwent PPV for ERM peeling and included all cases with sufficient data on (1) pre- and postoperative spectral domain optical coherence tomography (OCT) measurements and (2) pre- and postoperative functional measurements, including best corrected visual acuity (BCVA). All patients experienced a decline in visual acuity associated with clinically significant ERM. Patients with the presence and/or absence of posterior vitreous detachment (PVD) were included.

Our exclusion criteria were diabetic tractional retinal detachment, significant fibrovascular proliferation, macular hole, manifest uveitis, history of retinal detachment, aphakia, history of perforating or non-perforating trauma, and any other intraocular surgery despite uncomplicated cataract surgery with posterior chamber lens implantation. Patients with insufficient data, follow-up of less than one month, or poor-quality images were excluded.

### 2.2. Ophthalmologic Examination

All patients underwent pre- and postsurgical comprehensive ophthalmological examinations, including BCVA in decimal, intraocular pressure measurement, slit-lamp biomicroscopy, lens status, dilated fundus examination, and grade classification of diabetic retinopathy. Furthermore, we documented the intravitreal therapy (IVT) of anti-vascular endothelial growth factor (VEGF) and corticosteroids prior to surgery, along with the duration of diabetes mellitus, HbA1c percentage, arterial hypertension, hypercholesterolemia, chronic kidney disease, and smoking status, if applicable.

### 2.3. Optic Coherence Tomography

Preoperatively and at each postoperative visit, all eyes underwent macular examination with a spectral domain OCT using the Heidelberg Engineering Spectralis (Heidelberg Engineering GmbH, Heidelberg, Germany). The specific scan protocol was a custom raster scan pattern with 37 sections (512 A-scans each) in a 30° × 20° field of view. ERM was defined by visible epiretinal membranes and disappearance of physiological foveal depression in preoperative macular morphology assessment by OCT examination. Diabetic macular edema was defined as the presence of intraretinal fluid (IRF) and/or subretinal fluid (SRF) associated with increased central retinal thickness on OCT.

The following parameters were chosen for further analysis:

Central Subfield Retinal Thickness

The standard settings for OCT recordings were a 20° × 20° volume scan: 49 sections at a distance of 122 μm. The average central subfield retinal thickness (CSRT) in µm was automatically calculated by the device software as the distance from the retinal pigment epithelium to the internal limiting membrane (ILM) at the highest point within a circle of 1 mm radius centered on the fovea. We further noted the thickness of the superior, inferior, temporal, and nasal subfields.

Macular volume

The macular volume (MV, in mm^3^) was extracted from the topographic map of the macular cube scan. The central subfield had an inner diameter of 1 mm. Furthermore, we analyzed the volume of the outer nine segments, covering a total area of 28.27 mm^2^, corresponding to a scan diameter of 6 mm. These volumetric measurements were used for both pre- and postsurgical comparisons. No additional correction for axial length was applied, since the OCT system automatically adjusts for this during image acquisition.

Qualitative OCT parameters

The presence of hyperreflective foci (HF), IRF, and SRF was assessed on a representative central horizontal scan through the fovea. HF were manually counted in each scan. Only HFs with the following morphologic characteristics were evaluated in order to exclude hard exudates and microaneurysms from the analysis: (1) reflectivity similar to that of the nerve fiber layer; (2) absence of back-shadowing; and (3) <30 μm diameter. IRF was defined as round, minimally reflective spaces within the neurosensory retina, located in the outer nuclear layer, inner nuclear layer, or ganglion cell layer. SRF was characterized by subfoveal neurosensory hyporeflective detachment due to fluid accumulation between the retina and the retinal pigment epithelium. The definitions for HF, IRF, and SRF were based on the criteria described by Panozzo et al. [8].

**Figure 1:**
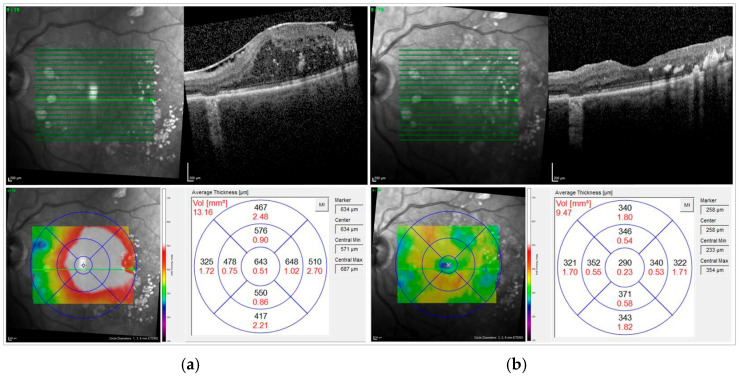
Exemplary case. Male, 80 years, CSRT: central subfield retinal thickness; ERM: epiretinal membrane, diabetes type 2 with secondary ERM (**a**) before and (**b**) 5 months after ERM peeling. Significant reduction in CSRT could be achieved. Visual acuity was 0.2 logMAR before surgery and did not improve (after surgery VA: 0.2 logMAR).

Figure 1 shows the OCT scan of an example case before and after surgery. 

### 2.4. Surgical Treatment

All patients underwent a three-port PPV, using either a 20-gauge or a sutureless 23- or 25-gauge technique, along with an ERM and ILM peeling. For staining of the ERM and ILM, Brilliant Peel^®^ (Geuder Company GmbH, Heidelberg, Germany) or Twin Blue (AL.CHI.MI.A. S.R.L., Ponte San Nicolò, Italy) was used at the surgeon’s discretion. The selected endotamponade options included air, sulfur-hexafluoride (20%), silicone oil (Siluron^®^ 2000 or 5000 cst; Geuder Company GmbH, Heidelberg, Germany), or balanced salt solution (BSS). For patients with concurrent cataracts, standard 2.2–2.8 mm clear cornea bimanual phacoemulsification was performed, followed by the implantation of a posterior chamber lens in the bag immediately before the PPV procedure. Additionally, any retinal breaks or degenerations identified during the surgery were treated with either laser retinopexy or cryoretinopexy. Furthermore, panretinal photocoagulation (PRP) or cryotherapy was performed in cases with severe NPDR or PDR at the discretion of the operating surgeon.

Postsurgical management included standard topical antibiotic and anti-inflammatory therapy, which was gradually tapered over at least two weeks. The procedures were performed by five different surgeons during the study period.

### 2.5. Main Outcome Measures

The primary outcome measures of this study were the functional assessment of BCVA and the morphological evaluation of CSRT and MV, analyzed at two time points: preoperatively and at the final postoperative follow-up. Secondary outcomes included the subgroup analysis of changes in BCVA in relation to perioperative lens status, DME, and stages of DR, as well as the subgroup analysis of qualitative OCT parameters in relation to both changes in BCVA and quantitative OCT metrics (CSRT and MV).

### 2.6. Statistical Methods

Statistical analysis was conducted using IBM SPSS Statistics for Windows, Version 26.0 (IBM Corp., Armonk, NY, USA). Data distribution was assessed using the Shapiro–Wilk test. Depending on distribution, paired *t*-tests were used for normally distributed variables, while the Wilcoxon signed-rank test was applied for non-normally distributed data. Descriptive statistics are reported as mean value ± standard deviation (SD). A *p*-value < 0.05 was considered statistically significant. Effect size was evaluated using Cohen’s d coefficient. As the focus was on changes within subjects rather than identifying independent predictors, no multivariable analysis was performed. To account for multiple comparisons, *p*-values were adjusted using the Bonferroni procedure to control the false discovery rate.

## 3. Results

### 3.1. Demographic Data and Clinical Characteristics

A total of 300 patients were initially screened for eligibility. Of these, 213 patients were excluded as they did not meet the inclusion criteria (IC) or fulfilled one or more of the exclusion criteria (EC). Consequently, 87 consecutive eyes of 87 patients were included in the final analysis. The mean age of the study population was 67.2 ± 10.2 years. The majority of patients were male (55/87, 63.2%), while 32/87 (36.8%) were female. The mean follow-up period was 26.3 ± 24.5 months (median 23.00 months). Regarding eye laterality, 36 eyes (41.4%) were right eyes, and 51 eyes (58.6%) were left eyes. The mean duration of diabetes mellitus (DM) was 19.8 ± 11.8 years. The majority of patients had type 2 DM (84/87, 96.6%); only three patients (3.4%) were diagnosed with type 1 DM. The mean HbA1c at baseline was 7.6 ± 1.1%, indicating moderate glycemic control across the cohort. Regarding lens status, 48 eyes (55.2%) were phakic and 39 eyes (44.8%) were pseudophakic at the time of surgery. DME was present in 56 patients (64.4%). Preoperative IVT for DME with either Anti-VEGF or corticosteroids was administered in 41 patients (47.1%).

Evaluation of the grade of DR showed that 11 patients (12.6%) had mild NPDR, moderate NPDR was observed in 17 patients (19.5%), and severe NPDR in 20 patients (23.0%). PDR was the largest subgroup, with 39 patients (44.8%). PRP was performed preoperatively in 63 eyes (mostly in patients with PDR, n = 37). Table 1 summarizes all baseline characteristics.

Out of the 87 patients, 6 underwent a combined procedure of ERM peeling and cataract surgery. Intraoperative PRP was required in 46.0% of patients, cryotherapy in 2.3%, and a combination of both in 5.7%. The need for intraoperative retinal laser or cryotherapy was predominantly observed in eyes with PDR and severe NPDR. Specifically, 29 patients with active PDR underwent PRP, and in four of these cases, additional cryoretinopexy was performed. In terms of intraoperative endotamponade selection, air was utilized in most cases, accounting for 68 eyes (78.2%). This was followed by sulfur-hexafluoride (SF6) 25% gas in 10 eyes (11.5%), silicone oil 5000 cst. (Siluron 5000^®^, Geuder AG, Heidelberg, Germany) in 5 eyes (5.7%), BSS in 3 eyes (3.4%), and silicone oil 2000 cst. (Siluron 2000^®^, Geuder AG, Heidelberg, Germany) in 1 eye (0.9%).

### 3.2. Functional Outcomes

In the overall cohort, BCVA did not show statistically significant changes following ERM peeling. Mean BCVA was 0.54 ± 0.39 logMAR preoperatively and 0.63 ± 0.66 logMAR at the last follow-up (*p* = 0.878; 26.3 ± 24.5 months).

When stratified by severity of diabetic retinopathy, no significant improvement in BCVA was observed in any subgroup. In eyes with mild NPDR, BCVA decreased from 0.37 ± 0.23 to 0.33 ± 0.28 logMAR (*p* = 0.591). In moderate NPDR, BCVA changed from 0.36 ± 0.15 to 0.45 ± 0.50 logMAR (*p* = 0.822). Severe NPDR eyes showed a slight insignificant decrease from 0.64 ± 0.45 to 0.62 ± 0.56 logMAR (*p* = 0.445), and in PDR, BCVA increased from 0.62 ± 0.43 to 0.80 ± 0.73 logMAR (*p* = 0.392).

Eyes with concurrent DME demonstrated a non-significant decline in BCVA from 0.64 ± 0.44 to 0.67 ± 0.58 logMAR postoperatively (*p* = 0.833). In contrast, eyes without DME showed a minor, non-significant decrease from 0.37 ± 0.16 to 0.55 ± 0.78 logMAR (*p* = 0.991).

No significant differences were observed in the comparison of subgroups based on lens status. In phakic eyes, BCVA changed from 0.51 ± 0.33 to 0.57 ± 0.62 logMAR (*p* = 0.710). In pseudophakic eyes, BCVA was 0.57 ± 0.45 at baseline and 0.71 ± 0.70 logMAR at last follow-up (*p* = 0.551).

Evaluation of qualitative parameters detected by OCT provided further insight. The presence of HF did not result in significant postoperative changes in BCVA (0.71 ± 0.53 to 0.65 ± 0.52 logMAR, *p* = 0.453). This was also observed in eyes without HF change (0.48 ± 0.29 to 0.63 ± 0.71 logMAR, *p* = 0.480).

Assessment of retinal fluid status showed that eyes without IRF or SRF exhibited a non-significant increase in BCVA postoperatively (0.37 ± 0.16 to 0.61 ± 0.82 logMAR, *p* = 0.605). Eyes with isolated IRF revealed a slight increase in BCVA postoperatively (0.61 ± 0.44 to 0.63 ± 0.59 logMAR, *p* = 0.861). Notably, eyes with both IRF and SRF demonstrated stable BCVA following surgery (0.72 ± 0.39 to 0.72 ± 0.50 logMAR, *p* = 0.713). Table 2 provides a detailed overview of these findings.

Postoperative visual acuity worsened in 28 cases compared to baseline. Reported causes included optic nerve atrophy, secondary cataract formation, persistence or progression of DME, worsening of DR with subsequent vitreous hemorrhage, and, in one case, a corneal ulcer with vascularization.

### 3.3. Anatomical Outcomes

Following ERM peeling, significant anatomical reduction was observed in both the thickness of the central retinal subfield and the macular volume. The mean CSRT decreased from 377.20 ± 99.28 µm preoperatively to 337.99 ± 113.83 µm postoperatively (adjusted *p* = 0.008). Similarly, the total MV showed a notable reduction, from 10.11 ± 1.46 mm^3^ before surgery to 9.28 ± 1.07 mm^3^ after the procedure (adjusted *p* < 0.001). The central MV also decreased significantly, from 0.33 ± 0.81 mm^3^ to 0.29 ± 0.69 mm^3^ (adjusted *p* < 0.001). The corresponding data is presented in Table 3.

#### Association Between Qualitative and Quantitative OCT Parameters

Quantitative OCT evaluation showed postoperative reductions in CSRT and MV in subgroups stratified by qualitative OCT characteristics.

In eyes with HF, CSRT decreased from 386.33 ± 120.01 µm to 303.25 ± 127.77 µm (Cohen’s d = 0.622, adjusted *p* = 0.003), while MV decreased from 9.57 ± 1.24 mm^3^ to 9.45 ± 1.19 mm^3^ (Cohen’s d = 0.698, adjusted *p* = 0.035). In eyes without HF, CSRT changed from 373.66 ± 90.88 µm to 352.44 ± 106.60 µm without significance, and MV changed from 10.11 ± 1.46 mm^3^ to 9.45 ± 1.19 mm^3^ (Cohen’s d = 0.792, adjusted *p* = 0.005).

Eyes without IRF or SRF showed an insignificant decrease in CSRT from 334.62 ± 87.71 µm to 320.52 ± 99.58 µm and in MV from 9.72 ± 1.22 mm^3^ to 9.01 ± 0.94 mm^3^. Eyes with IRF but without SRF demonstrated a significant CSRT reduction from 388.04 ± 96.91 µm to 336.89 ± 117.22 µm (Cohen’s d = 0.415, adjusted *p* = 0.020) and a significant MV reduction from 10.42 ± 1.38 mm^3^ to 9.41 ± 1.09 mm^3^ (Cohen’s d = 0.746, adjusted *p* = 0.005).

In the subgroup with both IRF and SRF, CSRT decreased insignificantly from 464.17 ± 98.90 µm to 426.50 ± 120.88 µm, and MV decreased from 11.80 ± 1.48 mm^3^ to 10.36 ± 1.65 mm^3^ (Cohen’s d 3.192, adjusted *p* = 0.04). These results are summarized in Table 4 and Table 5.

### 3.4. Subgroup Analysis of Anatomical and Functional Outcome

**Figure 2:**
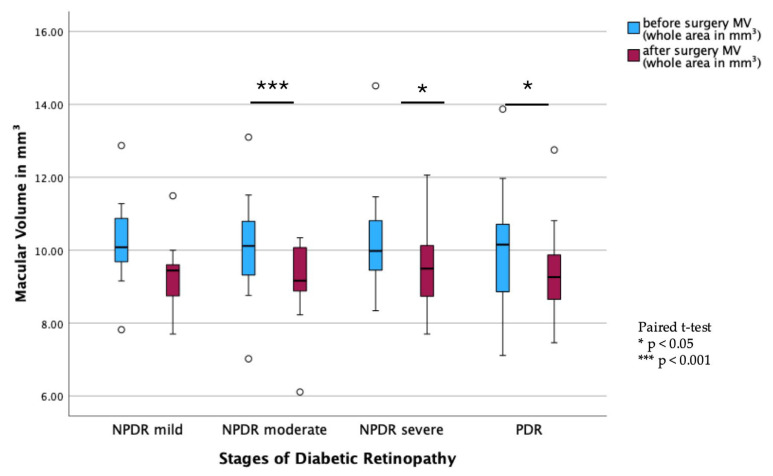
Macular volume (MV) for diabetic retinopathy (DR) subgroups. Boxplot illustrating pre- and postoperative MV in eyes with varying stages of DR (without DR, mild NPDR, moderate NPDR, severe NPDR, and PDR). Boxplots display the interquartile range (IQR), with the line indicating the median. Dots represent outliers (>1.5 × IQR). Paired *t*-test.

**Figure 3:**
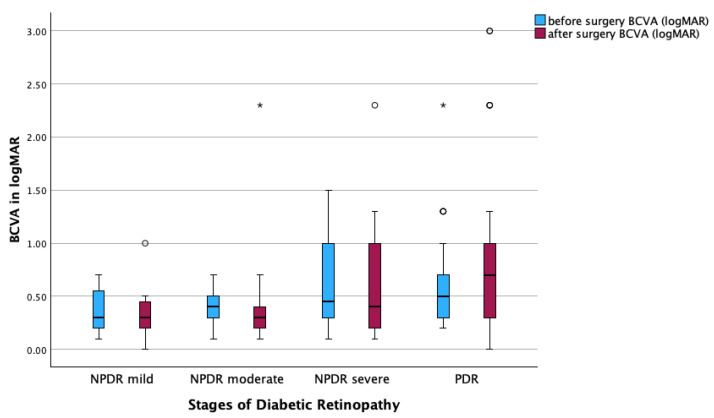
Functional results (BCVA) for diabetic retinopathy (DR) subgroups. Boxplot showing pre- and postoperative best-corrected visual acuity (BCVA; logMAR) across different stages of DR (without DR, mild NPDR, moderate NPDR, severe NPDR, and PDR). Boxplots display the interquartile range (IQR), with the line indicating the median. Dots represent outliers (>1.5 × IQR), and asterisks extreme outliers (>3 × IQR). Wilcoxon test.

Subgroup analysis of DR stages showed a significant decrease in MV from pre- to postoperative measurements in all stages of DR, except in the mild NPDR group (PDR: *p* < 0.001; moderate NPDR: *p* = 0.003; severe NPDR: *p* = 0.028; Figure 2). In contrast, there were no statistically significant changes in BCVA before and after surgery across all DR subgroups (Figure 3).

**Figure 4:**
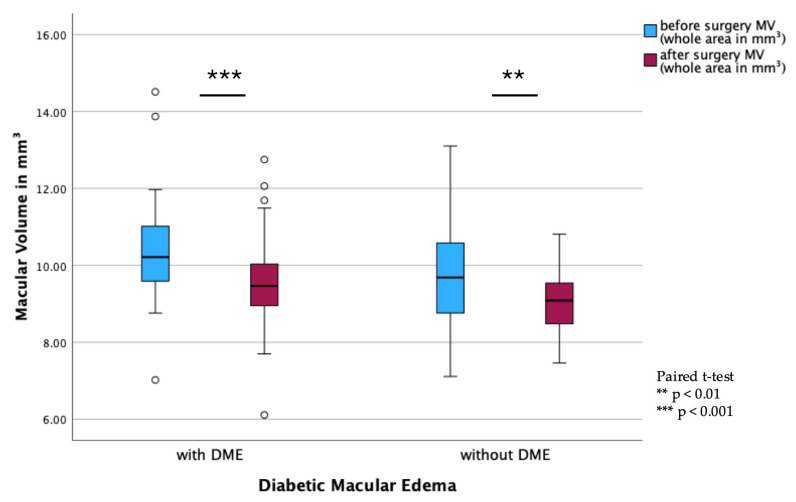
Macular volume (MV) in eyes with and without diabetic macular edema (DME). Boxplot displaying pre- and postoperative MV in eyes stratified by the presence or absence of DME. Boxplots present the interquartile range (IQR), with the line indicating the median. Dots represent outliers (>1.5 × IQR). Paired *t*-test.

**Figure 5:**
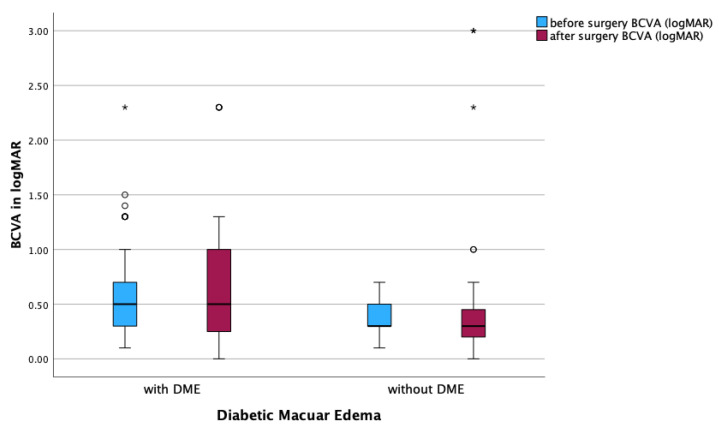
Functional results (BCVA) in eyes with and without diabetic macular edema (DME). Boxplot comparing pre- and postoperative best-corrected visual acuity (BCVA; logMAR) before and after surgery in patients with and without DME. Boxplots display the interquartile range (IQR), with the line indicating the median. Dots represent outliers (>1.5 × IQR), and asterisks extreme outliers (>3 × IQR). Wilcoxon test.

In the DME subgroups, a significant reduction in MV was observed in both DME (*p* < 0.001) and non-DME patients (*p* = 0.002; Figure 4). However, BCVA remained stable postoperatively, with no significant differences observed between pre- and postoperative values (Figure 5).

### 3.5. Complications

During the follow-up period, postoperative vitreous hemorrhage occurred in 2 of the 87 patients. One patient required reoperation 6 months after the initial procedure and subsequently developed a retinal detachment 13 months later, necessitating additional surgery. Among the phakic patients at baseline, 72.9% (35 out of 48 patients) required cataract surgery within 14.6 ± 21.2 months following ERM peeling. Postoperative OCT imaging revealed persistent diabetic macular edema (DME) in 85.0% of cases (51 of 60).

## 4. Discussion

Our study demonstrates significant anatomical improvements following vitrectomy with ERM peeling in patients with diabetic retinopathy, with a reduction in CSRT and MV. However, functional outcomes, as measured by BCVA, remained stable over time without significant long-term improvement. These findings suggest that, although anatomical restoration is achievable, sustained functional benefits may be limited by the progressive nature of diabetic retinal disease.

In our cohort, no significant improvements in BCVA were observed postoperatively. In patients with mild NPDR, there was a slight trend towards visual improvement, though it did not reach statistical significance. In contrast, eyes with more advanced stages of DR did not demonstrate visual rehabilitation. These findings differ from those reported by previous studies, which showed significant improvement in BCVA in diabetic patients who underwent ERM peeling [4,6,9,10]. A systematic review by Scheerlinck et al. focusing on patients with idiopathic ERM identified preoperative visual acuity as the most reliable predictive factor of postoperative visual acuity [11]. The absence of visual improvement in eyes with advanced DR may be attributed to irreversible retinal damage. In proliferative DR, chronic ischemia and widespread microvascular degeneration result in structural alterations such as photoreceptor loss, inner retinal layer damage, and disruption of the neurovascular unit [12]. While ERM peeling alleviates mechanical traction, it does not address these underlying degenerative pathologies. Moreover, advanced DR is frequently accompanied by macular ischemia and capillary dropout, which impair retinal perfusion and significantly limit the potential for postoperative visual recovery. Consequently, despite anatomical improvements, functional gains are unlikely in the presence of extensive neurovascular damage [12,13].

Our analysis showed significant reductions in CSRT, total MV, and central MV postoperatively, which align with previous studies reporting anatomical improvements following ERM removal in eyes with DME [4,9,10].

Previous reports suggested that eyes with advanced fibrovascular proliferation may particularly benefit from the relief of tractional forces achieved via ILM peeling during PPV with a reduction of secondary ERM formation in comparison to a PDR group without ILM peeling [14]. Furthermore, Le et al. published that the incidence of ERM formation following PRP was higher in patients with PDR (10.1%) compared to those with NPDR (5.7%), although this difference did not reach statistical significance [15]. Despite the lack of statistical significance, this trend suggests a greater predisposition for ERM development in advanced stages of DR. This is consistent with our findings, where the PDR subgroup represented the largest portion of the cohort and had frequently undergone PRP prior to surgery (37 of 39 eyes with PDR).

The role of DME in surgical outcomes remains complex. Regarding OCT parameters, significant anatomical recovery was observed in eyes with and without DME after ERM peeling, including significant reductions in central retinal thickness and macular volume. However, eyes with DME showed no significant improvement in BCVA following surgery, which is consistent with the previous literature noting the chronic impact of DME on visual recovery [9]. In contrast to our findings, Rabina et al. reported significant BCVA improvement following ERM peeling in patients with DME [4]. This discrepancy may be attributed to the smaller sample size in their study (n = 23) and the high proportion of combined procedures, with cataract surgery performed in 43.5% of cases, which potentially may have contributed to the observed functional gains.

Although visual acuity improvements in patients without DME did not reach statistical significance, the overall trend suggests that the absence of chronic intraretinal fluid preserves retinal microstructure and facilitates better postoperative healing. These findings support the hypothesis that patients without DME may have a more favorable visual prognosis after ERM surgery due to less chronic retinal damage and better-preserved tissue integrity [4].

Lens status did not influence the visual outcomes. We could not show significant improvements in both phakic and pseudophakic eyes. These findings are in contrast with Karger et al., who emphasized that while lens status influences visual rehabilitation, ERM removal independently contributes to visual improvement regardless of lens status [16].

Postoperative analysis across subgroups defined by qualitative OCT parameters revealed distinct anatomical and functional patterns. Eyes without IRF or SRF demonstrated a decrease in BCVA alongside non-significant reductions in CSRT and MV. Although the anatomical results were not statistically significant, a medium effect size was observed. The limited functional response to surgery, in the absence of retinal fluid, possibly reflects structural damage that is not responsive to surgical intervention.

Eyes with IRF alone also showed anatomical improvements, particularly in CSRT and MV, while BCVA remained largely unchanged. This is consistent with previous studies, which demonstrated that eyes with secondary ERM due to diabetes exhibited more persistent intraretinal cysts and enlarged capillary-free zones on OCT angiography after ERM peeling compared to idiopathic cases [17]. These changes have been attributed to Müller cell dysfunction and chronic retinal damage, potentially limiting visual recovery despite anatomical success [17].

In eyes presenting with both IRF and SRF, reductions in CSRT and MV were noted, but BCVA tended to remain largely stable despite lower baseline visual acuity. Although based on a limited number of cases, the combination of IRF and SRF may indicate more advanced retinal pathologies and a poorer capacity for functional recovery following ERM peeling.

Regarding the presence of HF, BCVA decreased after surgery. Significant anatomical improvements were observed in MV and CSRT. These findings support the hypothesis that HF may reflect chronic retinal inflammation or microglial activation, which could limit visual recovery, particularly in eyes with DME, as suggested in previous studies [18,19,20]. The group of patients without HF also showed similar visual outcomes but anatomical benefits after surgery.

Overall, the presence of IRF, SRF, and HF on OCT may indicate underlying retinal alterations that limit functional recovery despite anatomical improvements after ERM peeling in diabetic eyes.

This study has some limitations that should be acknowledged. First, the retrospective design and relatively limited sample size may affect the generalizability of our findings. Second, although we collected data on the duration of diabetes and glycemic control, other potential confounding factors—such as the timing and frequency of adjunctive treatments (e.g., IVF) and the presence of systemic comorbidities—were not fully controlled. Furthermore, we were unable to reliably assess the presence of ischemic maculopathy due to insufficient availability of preoperative fluorescein angiography or OCT angiography data. Additionally, the variability in follow-up duration across patients may have influenced the consistency of long-term visual outcome assessments. For patients who underwent surgery on both eyes, we selected the first-operated eye for analysis, as it typically represents the more severe case and has a longer follow-up period. We did not assess the presence or extent of posterior vitreous detachment using OCT, as this could not be reliably evaluated retrospectively in all cases using the available imaging data. Lastly, while significant anatomical improvements were observed, the relationship between these changes and functional visual recovery remains complex and warrants further investigation. Our findings underscore the multifactorial nature of visual outcomes in DR and the need for comprehensive management of both ocular and systemic comorbidities. Due to the retrospective nature of the study, no predictive conclusions regarding specific OCT features can be drawn. Further prospective studies with larger cohorts, additional imaging techniques (e.g., fluorescein angiography or OCT angiography), and standardized follow-up protocols are required to identify structural biomarkers associated with visual outcomes and to improve our understanding of the factors influencing visual and anatomical outcomes in this patient population.

## 5. Conclusions

Epiretinal membrane peeling in eyes with secondary ERM due to diabetes appears to be a safe and effective procedure for improving macular anatomy, as evidenced by significant reductions in CSRT and MV. However, despite these anatomical improvements, functional outcomes as measured by BCVA remain largely unchanged in the long term. These results suggest that visual recovery may be limited in the context of diabetic retinal disease, particularly in the presence of advanced DR, DME, or OCT signs such as IRF, SRF, and HF. Careful preoperative assessment is essential to identify patients who may benefit most from surgical intervention.

## Figures and Tables

**Table 1:** Baseline demographic and clinical characteristics.

Parameter	Total Cohort (n = 87)
Age (years; mean ± SD)	67.2 ± 10.2
Gender: male (%)	63.2 (n = 55)
Left eye (%)	58.6 (n = 51)
DM Duration (years; mean ± SD)	19.8 ± 11.8
DM Type 1 (%)	3.4 (n = 3)
DM Type 2 (%)	96.6 (n = 84)
HbA1c (%; mean ± SD)	7.6 ± 1.1
Lens Status: Phakic (%)	55.2 (n = 48)
Lens Status: Pseudophakic (%)	44.8 (n = 39)
Preoperative IVT (%)	47.1 (n = 41)
Anti-VEGF (%)	29.9 (n = 26)
Corticosteroids (%)	4.6 (n = 4)
Preoperative PRP (%)	72.4 (n = 63)
DME (%)	69.0 (n = 60)
Stages of Diabetic Retinopathy	
NPDR mild (%)	12.6 (n = 11)
NPDR moderate (%)	19.5 (n = 17)
NPDR severe (%)	23.0 (n = 20)
PDR (%)	44.8 (n = 39)

Summary of patient demographics, ocular findings, and diabetes-related parameters at baseline. Continuous variables are expressed as mean ± standard deviation, and categorical variables as percentages. DME: diabetic macular edema; DM: diabetes mellitus; DR: diabetic retinopathy; HbA1c: glycated hemoglobin; IVT: intravitreal therapy (exact agent unspecified in 11 cases; corticosteroids used were either triamcinolone or Ozurdex); NPDR: non-proliferative diabetic retinopathy; PDR: proliferative diabetic retinopathy; PRP: panretinal photocoagulation; SD: standard deviation; and VEGF: vascular endothelial growth factor.

**Table 2:** Changes in best-corrected visual acuity before and after ERM peeling in patients with diabetic retinopathy.

Study Cohort	BCVA (logMAR; Mean ± SD) Before ERM Peeling	BCVA (logMAR; Mean ± SD) After ERM Peeling (Last Follow-Up; Mean 26.3 Months)	*p*-Value ^1^
Overall Cohort (n = 87)	0.54 ± 0.39	0.63 ± 0.66	0.878
Stages of Diabetic Retinopathy			
NPDR mild (n = 11)	0.37 ± 0.23	0.33 ± 0.28	0.591
NPDR moderate (n = 17)	0.36 ± 0.15	0.45 ± 0.50	0.822
NPDR severe (n = 20)	0.64 ± 0.45	0.62 ± 0.56	0.445
PDR (n = 39)	0.62 ± 0.43	0.80 ± 0.79	0.392
Diabetic Macular Edema			
With DME (n = 60)	0.64 ± 0.44	0.67 ± 0.58	0.833
Without DME (n = 27)	0.37 ± 0.16	0.55 ± 0.78	0.991
Lens Status			
Phakic (n = 48)	0.51 ± 0.33	0.57 ± 0.62	0.710
Pseudophakic (n = 39)	0.57 ± 0.45	0.71 ± 0.70	0.551
Qualitative OCT Parameters			
Presence of HF (n = 24)	0.71 ± 0.53	0.65 ± 0.52	0.453
Absence of HF (n = 62)	0.48 ± 0.29	0.63 ± 0.71	0.480
Absence of IRF or SRF (n = 27)	0.37 ± 0.16	0.61 ± 0.82	0.605
Presence of IRF, absence of SRF (n = 54)	0.61 ± 0.44	0.63 ± 0.59	0.861
Presence of IRF and SRF (n = 6)	0.72 ± 0.39	0.72 ± 0.50	0.713

^1^ Wilcoxon test. BCVA: best-corrected visual acuity; DME: diabetic macular edema; ERM: epiretinal membrane; HF: hyperreflective foci; IRF: intraretinal fluid; logMAR: logarithm of the minimum angle of resolution; NPDR: non-proliferative diabetic retinopathy; PDR: proliferative diabetic retinopathy; SD: standard deviation; and SRF: subretinal fluid.

**Table 3:** Anatomical outcomes in central subfield retinal thickness and macular volume following ERM peeling.

OCT Parameters	Before ERM Peeling	After ERM Peeling (Last Follow-up; Mean: 26.3 Months)	Adjusted *p*-Value ^1^
Central Subfield Retinal Thickness (µm; mean ± SD)	377.20 ± 99.28	337.99 ± 113.83	**0.008**
Macular Volume (mm^3^; mean ± SD)	Total 10.11 ± 1.46	9.28 ± 1.07	**<0.001**
	Central 0.33 ± 0.81	0.29 ± 0.69	**<0.001**

^1^ Paired *t*-test. *p*-values have been adjusted for multiple comparisons using the Bonferroni procedure. ERM: epiretinal membrane; OCT: optical coherence tomography.

**Table 4:** Correlation analysis between changes in central subfield retinal thickness and qualitative OCT parameters.

Total Cohort (n = 87)	CSRT in µm Before ERM Peeling (Mean ± SD)	CSRT in µm (Mean ± SD) After ERM Peeling (Last Follow-Up; Mean: 26.3 Months)	Cohen’s d	Adjusted *p*-Value ^1^
Qualitative OCT Parameters				
Presence of HF (n = 24)	386.33 ± 120.01	303.25 ± 127.77	0.622	**0.003**
Absence of HF (n = 62)	373.66 ± 90.88	352.44 ± 106.60	0.221	0.435
Absence of IRF or SRF (n = 26)	334.62 ± 87.71	320.52 ± 99.58	0.698	1.000
Presence of IRF, absence of SRF (n = 54)	388.04 ± 96.91	336.89 ± 117.22	0.415	**0.020**
Presence of IRF and SRF (n = 6)	464.17 ± 98.90	426.50 ± 120.88	0.812	0.515

^1^ paired *t*-test. *p*-values have been adjusted for multiple comparisons using the Bonferroni procedure. CSRT: central subfield retinal thickness; ERM: epiretinal membrane; HF: hyperreflective foci; IRF: intraretinal fluid; OCT: optical coherence tomography; SD: standard deviation; and SRF: subretinal fluid.

**Table 5:** Correlation analysis between changes in macular volume and qualitative OCT parameters.

Total Cohort (n = 87)	MV in mm^3^ Before ERM Peeling (Mean ± SD)	MV in mm^3^ (Mean ± SD) After ERM Peeling (Last Follow-up; Mean: 26.3 months)	Cohen’s d	Adjusted *p*-Value ^1^
Qualitative OCT Parameters				
Presence of HF (n = 19)	10.51 ± 1.34	9.45 ± 1.19	0.698	**0.035**
Absence of HF (n = 47)	10.11 ± 1.46	9.28 ± 1.07	0.792	**0.005**
Absence of IRF or SRF (n = 22)	9.57 ± 1.24	9.01 ± 0.94	0.571	0.070
Presence of IRF, absence of SRF (n = 40)	10.42 ± 1.38	9.41 ± 1.09	0.746	**0.005**
Presence of IRF and SRF (n = 4)	11.80 ± 1.48	10.36 ± 1.65	3.192	**0.04**

^1^ paired *t*-test. *p*-values have been adjusted for multiple comparisons using the Bonferroni procedure. MV: macular volume; ERM: epiretinal membrane; HF: hyperreflective foci; IRF: intraretinal fluid; OCT: optical coherence tomography; SD: standard deviation; and SRF: subretinal fluid.

## Data Availability

The datasets generated during and/or analyzed during the current study are available from the corresponding author upon reasonable request.

## Abbreviations

The following abbreviations are used in this manuscript:

BCVA	Best-corrected visual acuity
BSS	Balanced salt solution
CSRT	Central subfield retinal thickness
DM	Diabetes mellitus
DME	Diabetic macular edema
DR	Diabetic retinopathy
EC	Exclusion criteria
ERM	Epiretinal membrane
HbA1c	Glycated hemoglobin
HF	Hyperreflective foci
IC	Inclusion criteria
ILM	Internal limiting membrane
IRF	Intraretinal fluid
IVT	Intravitreal therapy
logMAR	Logarithm of the minimum angle of resolution
MV	Macular volume
NPDR	Non-proliferative diabetic retinopathy
OCT	Optical coherence tomography
PDR	Proliferative diabetic retinopathy
PRP	Panretinal photocoagulation
PPV	Pars plana vitrectomy
SD	Standard deviation
SD-OCT	Spectral domain optical coherence tomography
SF6	Sulfur-hexafluoride
SRF	Subretinal fluid
VEGF	Vascular endothelial growth factor

## References

[1] Ghazi-Nouri S.M.S., Tranos P.G., Rubin G.S., Adams Z.C., Charteris D.G. (2006). Visual Function and Quality of Life Following Vitrectomy and Epiretinal Membrane Peel Surgery. Br. J. Ophthalmol..

[2] Fung A.T., Galvin J., Tran T. (2021). Epiretinal Membrane: A Review. Clin. Experiment. Ophthalmol..

[3] Hecht I., Karesvuo M., Kanclerz P., Jeon S., Karesvuo P., Tuuminen R. (2024). The Effect of Diabetes on Short-Term Outcomes Following Epiretinal Membrane Surgery. Int. Ophthalmol..

[4] Rabina G., Hilely A., Barequet D., Cohen S., Lipin N., Mimouni M., Glick A., Barak A., Loewenstein A., Schwartz S. (2022). Epiretinal Membrane in Patients with Diabetic Macular Edema. Ophthalmologica.

[5] Kang Y.K., Park H.S., Park D.H., Shin J.P. (2020). Incidence and Treatment Outcomes of Secondary Epiretinal Membrane Following Intravitreal Injection for Diabetic Macular Edema. Sci. Rep..

[6] Ozturk M., Guven D., Kacar H., Karapapak M., Demir M. (2021). Functional and Morphological Results of Epiretinal Membrane Surgery in Idiopathic versus Diabetic Epiretinal Membranes. Semin. Ophthalmol..

[7] Wilkinson C.P., Ferris F.L., Klein R.E., Lee P.P., Agardh C.D., Davis M., Dills D., Kampik A., Pararajasegaram R., Verdaguer J.T. (2003). Proposed International Clinical Diabetic Retinopathy and Diabetic Macular Edema Disease Severity Scales. Ophthalmology.

[8] Panozzo G., Cicinelli M.V., Augustin A.J., Battaglia Parodi M., Cunha-Vaz J., Guarnaccia G., Kodjikian L., Jampol L.M., Jünemann A., Lanzetta P. (2020). An Optical Coherence Tomography-Based Grading of Diabetic Maculopathy Proposed by an International Expert Panel: The European School for Advanced Studies in Ophthalmology Classification. Eur. J. Ophthalmol..

[9] Flaxel C.J., Edwards A.R., Aiello L.P., Arrigg P.G., Beck R.W., Bressler N.M., Bressler S.B., Ferris F.L., Gupta S.K., Haller J.A. (2010). Factors Associated with Visual Acuity Outcomes after Vitrectomy for Diabetic Macular Edema: Diabetic Retinopathy Clinical Research Network. Retina.

[10] Yüksel K., Karaküçük Y., Özkaya A., Pekel G., Baz Ö., Alagöz C., Yazıcı A.T. (2015). Comparison of Photoreceptor Outer Segment Length in Diabetic and Idiopathic Epiretinal Membranes. Eye.

[11] Scheerlinck L.M.E., van der Valk R., van Leeuwen R. (2015). Predictive Factors for Postoperative Visual Acuity in Idiopathic Epiretinal Membrane: A Systematic Review. Acta Ophthalmol..

[12] Homme R.P., Singh M., Majumder A., George A.K., Nair K., Sandhu H.S., Tyagi N., Lominadze D., Tyagi S.C. (2018). Remodeling of Retinal Architecture in Diabetic Retinopathy: Disruption of Ocular Physiology and Visual Functions by Inflammatory Gene Products and Pyroptosis. Front. Physiol..

[13] Sim D.A., Keane P.A., Zarranz-Ventura J., Fung S., Powner M.B., Platteau E., Bunce C.V., Fruttiger M., Patel P.J., Tufail A. (2013). The Effects of Macular Ischemia on Visual Acuity in Diabetic Retinopathy. Invest. Ophthalmol. Vis. Sci..

[14] Wu R.-H., Xu M.-N., Lin K., Ren M.-X., Wen H., Feng K.-M., Zhou H.-J., Moonasar N., Lin Z. (2022). Inner Limiting Membrane Peeling Prevents Secondary Epiretinal Membrane after Vitrectomy for Proliferative Diabetic Retinopathy. Int. J. Ophthalmol..

[15] Le P., Nguyen M., Lee K., Vu T., George R., Landers M.B., Zhang A.Y. (2025). Incidence of Epiretinal Membrane Formation Following Treatment of Diabetic Retinopathy with Panretinal Photocoagulation Therapy. Int. J. Ophthalmol..

[16] Chatzistergiou V., Papasavvas I., Ambresin A., Pournaras J.-A.C. (2021). Prediction of Postoperative Visual Outcome in Patients with Idiopathic Epiretinal Membrane. Ophthalmologica.

[17] Romano M.R., Ilardi G., Ferrara M., Cennamo G., Allegrini D., Pafundi P.C., Costagliola C., Staibano S., Cennamo G. (2018). Intraretinal Changes in Idiopathic versus Diabetic Epiretinal Membranes after Macular Peeling. PLoS ONE.

[18] Von Schulthess E.L., Maunz A., Chakravarthy U., Holekamp N., Pauleikhoff D., Patel K., Bachmeier I., Yu S., Cohen Y., Scherb M.P. (2024). Intraretinal Hyper-Reflective Foci Are Almost Universally Present and Co-Localize With Intraretinal Fluid in Diabetic Macular Edema. Invest. Ophthalmol. Vis. Sci..

[19] Huang H., Jansonius N.M., Chen H., Los L.I. (2022). Hyperreflective Dots on OCT as a Predictor of Treatment Outcome in Diabetic Macular Edema: A Systematic Review. Ophthalmol. Retina.

[20] Tang L., Luo D., Qiu Q., Xu G.-T., Zhang J. (2023). Hyperreflective Foci in Diabetic Macular Edema with Subretinal Fluid: Association with Visual Outcomes after Anti-VEGF Treatment. Ophthalmic Res..

